# Dysbiosis in the Family nucleus of Children Diagnosed With Autism Spectrumin Mexico City

**DOI:** 10.62641/aep.v54i1.1986

**Published:** 2026-02-15

**Authors:** Alma Delia Genis Mendoza, Lucero Nuncio-Mora, Venancio Sánchez, Vanessa Gonzalez, Humberto Nicolini

**Affiliations:** ^1^Dr. Juan N. Navarro Children’s Psychiatric Hospital, CONASAMA, Secretaria de Salud Secretary of Health, 14080 Mexico city, Mexico; ^2^Laboratory of Genomics of Psychiatric and Degenerative Diseases, National Institute of Genomic Medicine, Ministry of Health, 14610 Mexico city, Mexico; ^3^Pharmacogenomics Laboratory, National Institute of Genomic Medicine, Ministry of Health, 14610 Mexico city, Mexico

**Keywords:** microbiota, parents autism, dysbiosis, Mexico

## Abstract

**Background:**

The relationship between the gut microbiome and Autism Spectrum Disorder (ASD) has been the subject of growing interest in scientific research. Research into the relationship between the gut microbiome and ASD has gained relevance in recent years as recent studies have identified significant differences in the gut microbiome abundance and composition in ASD children compared to neurotypical ones. However, little is known about the microbiome interplay, changes and relationship in parents and children with ASD, considering that they share a consistent environment. Charactering the microbiota of trio-type families with a child diagnosed with autism.

**Methods:**

The hypervariable region of the 16s ribosomal gene was sequenced from stool samples from adolescents with ASD and their parents. The analysis was performed using various software programs, including QIIME2 and DADA2.

**Results:**

In this paper, we discuss this relationship in three families, and observed that the gut microbiome of the offspring with ASD is more similar to the mother's than the father's microbiome.

**Conclusions:**

These observations could lead to the understanding of the potential heritability of the disorder through parental connectedness of the gut microbiome and eventually to the development of interventions aimed at modulating the gut microbiota to improve symptoms associated with ASD.

## Introduction

The relationship between the gut microbiome and Autism Spectrum Disorder (ASD) has been the subject of increasing interest in scientific research [1, 2]. Recent studies have identified significant differences in the composition of the gut microbiome of children with ASD compared to neurotypical children [1, 3]. For example, alterations have been found in bacteria such as *Bacteroides*, *Lachnospira*, *Anaerobutyricum* and *Ruminococcus torques*, which could be associated with autism. In addition, it has been observed that many children with ASD present gastrointestinal problems, such as constipation and diarrhea, suggesting a connection between the gut microbiota and gastrointestinal symptoms in autism [2, 4, 5, 6]. Research has also explored interventions aimed at modifying the gut microbiota to alleviate ASD symptoms. For example, a study published in 2019 investigated the effects of microbiota transfer therapy (MTT) in individuals with ASD [7]. The results indicated that MTT altered the gut ecosystem and improved gastrointestinal and autism-related symptoms [7, 8].

It is important to note that not all studies have found a direct causal relationship between gut microbiota and autism. One study published in July 2024 concluded that there was no connection between autism and the content of the gut microbiome. The exact nature of this relationship remains complex and requires further research to fully understand its implications [9].

In Mexico, research on this topic has been little addressed [10], although investigations have highlighted the importance of understanding how alterations in the intestinal microbiota can influence ASD symptoms. A study published in July 2023 introduced a two-step single-plex polymerase chain reaction (PCR) method to assess key markers of the colonic microbiota in Mexican youth with ASD. This pilot epidemiological application aimed to identify specific microbiota markers associated with ASD in the Mexican population [11]. Variation in intestinal microbial populations is associated with an increased risk of gastrointestinal symptoms such as chronic constipation and diarrhea, which can decrease quality of life [12]. It is essential to continue research in the field to develop therapeutic interventions to modulate the intestinal microbiota and improve the quality of life of people with ASD. These observations could lead to the understanding of the potential heritability of the disorder through parental connectedness of the gut microbiome and eventually to the development of interventions aimed at modulating the gut microbiota to improve symptoms associated with ASD in Mexico.

## Methods

### Study Participants

Nine parent-offspring, were recruited in December 2018 in Mexico City, i.e., 3 ASD children and they 2 parents, ASD children were aged 5, 10, and 13 years, and parents were between 38 and 44 years.

Patients were evaluated and diagnosed by a specialized psychiatrist. Inclusion criteria for children with autism were patients aged 5 to 15 years, who met the criteria for ASD. All children were assessed for ASD using the M-CHAT scale [13]. The M-CHAT has 23 questions, each with a score of 0 or 1 for all items except items 2, 5, and 12. A response of “No” indicates a high risk for ASD. For items 2, 5, and 12, a response of “Yes” indicates a high risk. No children were excluded if they failed more than two critical items or more than three items on the M-CHAT scale. If the score is greater than 0–3, the risk is low. If it’s between 4–7, the risk is medium. And if it’s higher than 8 points, the risk is high, the risk is high. In the case of children in the ASD group, all children were assessed by an expert child psychiatrist and a board-certified child psychiatrist. ASD diagnosis was based on Diagnostic and Statistical Manual of Mental Disorders (DSM-IV) criteria and was confirmed using the Revised Autism Diagnostic Interview (ADI-R) instrument [14]. Not having used antibiotics for at least 3 months prior to stool sampling and not having performed surgical procedures such as gastroscopy and colonoscopy (in the last 3 months) or any major gastrointestinal surgery for at least 5 years. The same criteria were used for parents. All participants signed an informed consent and assent form, as appropriate. The project was reviewed and approved by the Research Ethics Committee of the “Dr. Juan N. Navarro” Children’s Psychiatric Hospital and the National Institute of Genomic Medicine (CONBIOETICA CI2015/49) in accordance with the Declaration of Helsinki. Each participant signed the informed consent or assent, as appropriate.

### Sample Collection and DNA Extraction

Fecal sample collection and DNA extraction were performed following the protocol previously described [15]. Briefly, participants collected stool samples at home and then were stored at 4 °C and delivered to the research team within 24–48 hours. Upon receipt, samples were aliquoted under sterile conditions and stored at –80 °C until processing.

DNA extraction was conducted using the QIAmpPowerFecal Pro Kit (Qiagen, Hilden, Germany) according to the manufacturer’s instructions. DNA purity and concentration were assessed using a NanoDrop 2000c spectrophotometer (ThermoFisher Scientific, Waltham, MA, USA), ensuring that the A260/280 ratio was within the acceptable range (1.8–2.0). DNA integrity was verified via 1% agarose gel electrophoresis.

### Amplification and Sequencing

The amplification of the V3-V4 region of the 16S rRNA gene and library preparation were performed as previously described in Nuncio-Mora *et al*. [15]. The 16S V3 (341F) forward and V4 (805R) reverse primers with Illumina adapters were used. Library quality control was assessed using microcapillary electrophoresis on a TapeStation 4200 (Agilent Technologies, Santa Clara, CA, USA). Libraries were then normalized, denatured, and diluted for sequencing.

Sequencing was performed on a MiSeq platform (Illumina, San Diego, CA, USA) using a MiSeq Reagent Kit V3 (2 × 250 bp) at the Sequencing Unit of the National Institute of Genomic Medicine (INMEGEN, Mexico).

### Bioinformatic Analyses

Raw sequencing data obtained from the MiSeq platform (Illumina, San Diego, CA, USA) were processed and analyzed using QIIME2 Quantitative Insights Into Microbial Ecology 2 (version 2024.5) [16], following the pipeline previously described in Nuncio-Mora *et al*. [15]. Paired-end reads (2 × 250 bp) were quality-filtered, and sequences with a quality score below 20 were truncated.

Denoising and chimera removal were performed using DADA2 (version 2024.5.0) [17]. Amplicon sequence variants (ASVs) were aligned with the MAFFT algorithm (version 2024.5.0) [18] and used to construct a phylogenetic tree.

Taxonomic assignment of amplicon sequence variants (ASVs) was performed using SILVA (version 138.2) database as a reference, pre-trained with the *classify-Sklearn* Naïve Bayes classifier (version 2024.5.0). Differential abundance analysis of microbial taxa was conducted using the MaAsLin2 package (version 1.14.1), adjusting for covariates using the formula: DX [15].

## Results

### Clinical Data

Microbiome analysis was performed using the sequences from the three children as a group to determine whether there were consistent groups of microorganisms in all three children.

### Sequencing Data

A total of 515,018 raw sequences were identified for both forward and reverse reads in 9 samples processed. After filtering and chimera removal, 230,798 amplicon sequence variants (ASVs) were obtained; the average number of ASVs per sample was 50,935 reads (min 26,561; max 62,328) and considered for further bioinformatic analyses in the Software QIIME2 (version 2024.5).

### Gut Microbiome Abundance on Parents and ASD Offspring

**Fig. 1.**
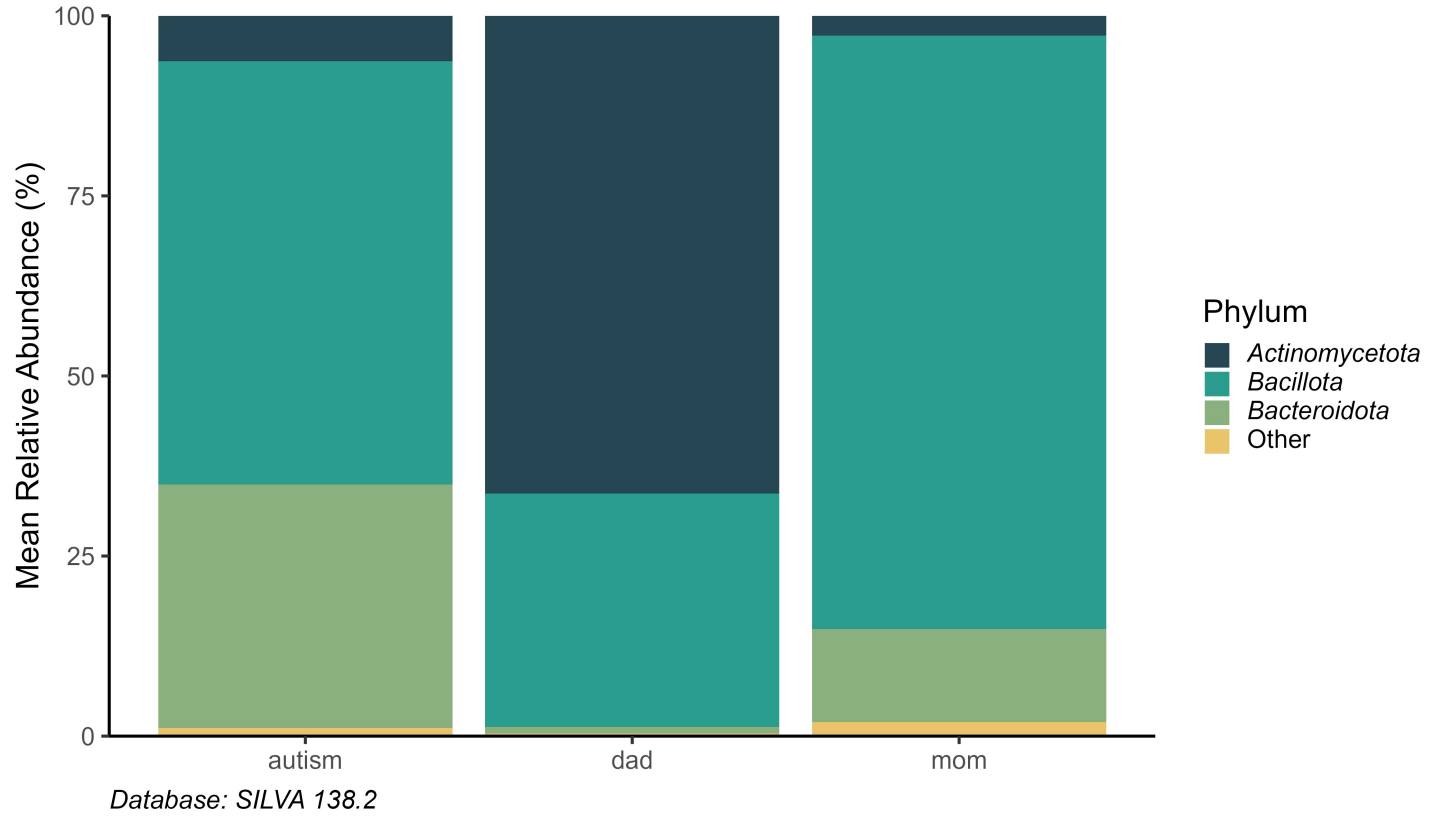
**Mean relative abundance of Phyla in parent-ASD offspring**. ASD, Autism Spectrum Disorder.

The Composition of the Gut Microbiome encompassed 8 phylumincluding in order of abundance, *Actinomycetota* (66.3%) was the most abundant phylum, followed by *Bacillota* (32.4%), and *Bacteroidota* (0.9%) in fathers (Fig. 1). In women, *Bacillota* (82.3%) was the most abundant phylum, followed by *Bacteroidota* (12.9%) and *Actinomycetota* (2.7%). For ASD children *Bacillota* (58.7%) was the most abundant phylum, followed by *Bacteroidota* (33.78%) and *Actinomycetota* (6.3%).

**Fig. 2.**
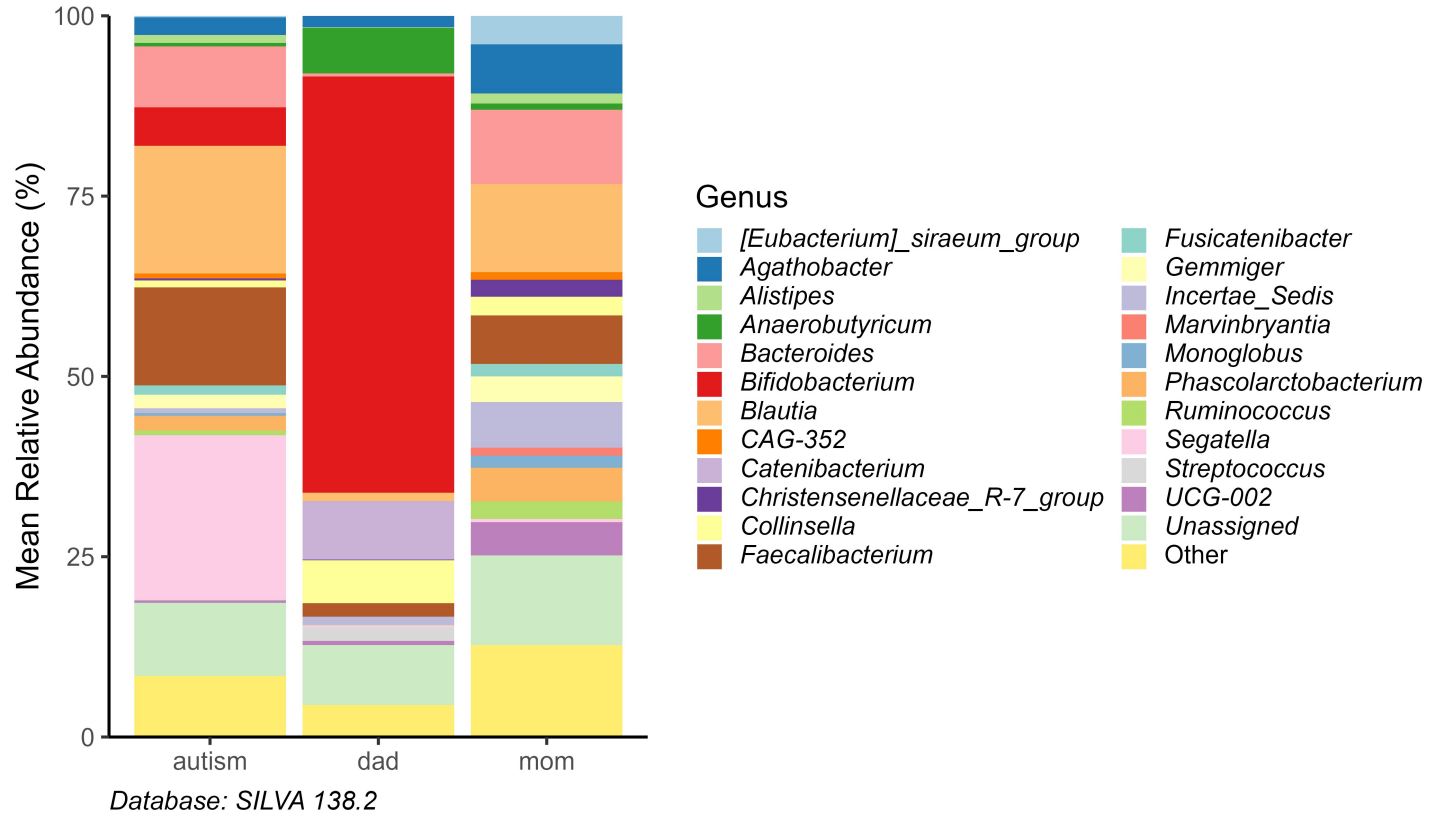
**Relative abundance at the genuslevel in parents and children with ASD**.

At the genus level in ASD children we identified 106 different genera of which 24 exhibited a relative abundance greater than 1% (Fig. 2).

The mean relative abundance was compared between parents and offspring a nivel genus and we observed that in ASD children there was a greater abundance in the genre *Segatella* (22.81%), *Blautia* (17.71%), *Faecalibacterium* (13.59%) and *Bacteroides* (8.47%). ASD children’s mothers, mostly presents *Blautia* (12.24%), *Bacteroides* (10.25%), *Agathobacter* (6.80%), and *Fecalibacterium* (6.69%). In contrast, fathers mostly presented *Bifidobacterium* (57.7%), *Catenibacterium* (8.07%), *Anaerobutyricum* (6.28%), *Collinsella* (5.9%), and *Fecalibacterium* (1.89%). We observe that fifteen genders were shared by parents and children with ASD, although their abundance was completely different.

**Fig. 3.**
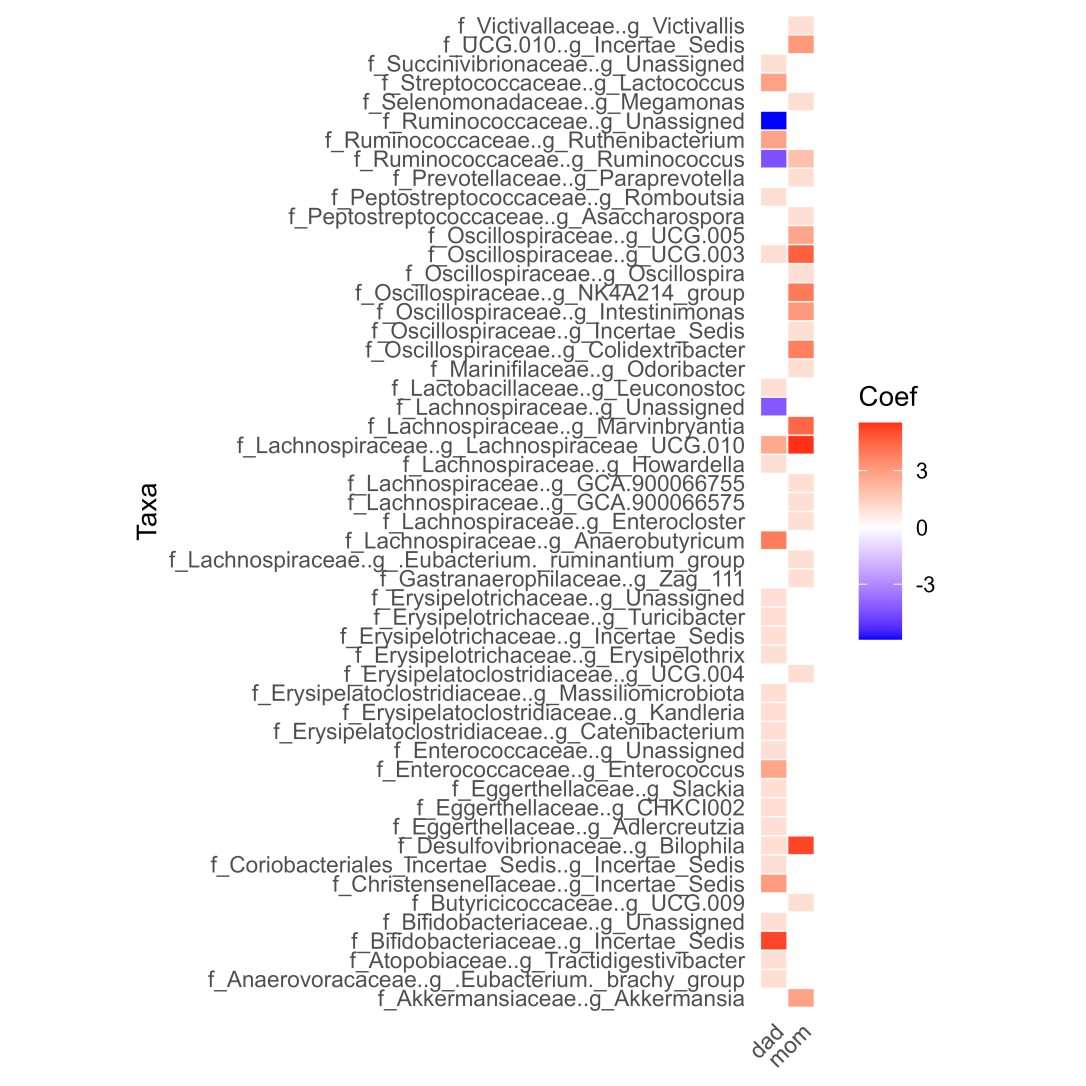
**The microbiome abundance of ASD patients compared with that of their parents**.

The Fig. 3, shows that mothers share more genders with their children. Unlike fathers, whose reported genders, despite living in the same house, are very different from those of their children.

## Discussion

The gut microbiota has been an area of great interest in autism research, as a connection has been found between gut health and neurological development. ASD patients often present an altered gut microbiome compared to individuals without autism. Lower microbial diversity has been observed in the ASD, which has been hypothesized to impair the immune system regulation and digestion. Group comparisons showed similarities at the phylum level with quite marked differences in the amount of bacteria, with *Bacillota* being the most abundant. Comparisons between groups showed some similarities at the phylum level, with quite marked differences in bacterial abundance, with *Bacillota* being the most abundant across groups.

We also observed the relative abundance and diversity of the gut microbiome of mothers and fathers of children with ASD. Regarding fathers, shows the relative abundance of the microbiota. Fathers of children with ASD showed lower relative diversity, with 10 genera. It is important to note that the fathers’ group showed the genusacillota (32.4%) and *Bacteroidota* (0.9%) as the most abundant in this group. In contrast, mothers showed a greater relative abundance and diversity, with the identification of 20 genera. *Bacillota* (82.3%) was the most abundant, followed by *Bacteroidota* (12.9%) and *Actinomycetota* (2.7%).

Interestingly, the ASD group had a lower relative abundanceas it agrees with the literature [12, 19] compared to the father and mother groups. In children with ASD, only 10 different genera were reported, with *Bacillota* (58.7%) being the most abundant phylum, followed by *Bacteroidota* (33.78%) and *Actinomycetota* (6.3%). *Segatella* was the most represented. Studies on ASD in Mexico are scarce, and the results are varied. A recent study from Mexico City, reported no significant differences in the dominant bacterial phyla (Firmicutes, *Bacteroidota*, Actinobacteria, Proteobacteria, Verrucomicrobiota) between the ASD and NT groups, but by genus, disparities were apparent for the abundance of *Blautia*, Prevotella, Clostridium XI and Clostridium XVIII, all of which have been previously associated with ASD [11]. In our analysis we identified the genus *Segatella*, represented only in ASD, further studies may define which species of *Segatella* are present in ASD patients and if these this could show clinical utility as a potential marker for patients also if *Segatella* species could be clinically useful only in Mexico or these observations may be expanded to other regions. In agreement, a 2024 study by Shao *et al*. [20], authors observed that children with ASD presented *Bifidobacterium* bifidum and Segatellacopri, and an increase in sphingolipid metabolism when compared to NT. Clostridium and Desulfovibrio were observed here as in children with ASD, these bacteria have been reported to be the most abundant genera in children with ASD. However, these were not observed here perhaps due to the small sample or local differences in the environment and diet. Genera with the lowest abundance in ASD children has been observed for Bifidobacterium, Agathobacter, Alistipes, and CAG-352, and was observed with menor abundance. It is well acknowledged that ASD children show a reduction in *Bifidobacterium* and Lactobacillus, with an increase in Clostridium, and Desulfovibrio, these changes have been associated with inflammation and digestive complications. In our analysis, we found that Bifidobacterium is one of the least represented genera, although Agathobacter, Alistipes, and CAG-352 were found also in lower abundance compared to children without ASD; with *Segatella*, *Blautia*, and *Faecalibacterium* being the genera with the greater abundance [1]. Although one of the reported genera *Segatella* is consistent with the literature, the results show that the difference between these genera is possibly due to the type of diet, which tends to be very selective in ASD, since diets between countries and regions are different.

In the association analysis, shared or different genera were compared between the microbiome of the fathers’ and mothers’ groups compared to those with ASD. The genera shared between the three groups, as Bifidibacteriaceae, Butyricicoccaceae UCG.009, and Erysipelotrichaceae Kngleria, were found to be more abundant and statistically significant. It is noteworthy that some genera, such as *Catenibacterium*, were found only in the fathers’ group, while Megamonas was the most abundant in the mothers’ group. It is well acknowledged that ASD children show a reduction in *Bifidobacterium* and Lactobacillus, with an increase in Clostridium, and Desulfovibrio, these changes have been associated with inflammation and digestive complications. There are few studies comparing the microbiota of fathers and mothers with respect to ASD. We only found one publication in China a 2019, study investigated the microbiota of a child with ASD and his mother, finding significant differences in the abundance of Alcaligenaceae and Acinetobacter. Mothers of children with ASD had a higher abundance of Proteobacteria, Alphaproteobacteria, Moraxellaceae, and Acinetobacter than mothers of neurotypical children [21].

Our observations are limited by the sample size and the lack of comparisons with other regions of the country hence it is not possible to discard that these differences may be influenced by environmental factors including local diet patterns [22]. Nevertheless, when considering comparisons among family members living together we may decrease the heterogeneity of this relationship, still we ought to consider that household members may differ in eating habits and patterns factors that will be considered in future studies [23]. Other external factors influencing our results may include individual stress, family interactions, and work conditions of employed parents. This was an exploratory analysis that hints towards closer similarities in the gut microbiome between mothers and ASD children when compared to their fathers. Future studies will focus on validating these results to confirm the relationship between the gut microbiome of ASD children and their parents.

## Conclusions

The microbiota of fathers’ groups is different from that of ASD, while the microbiome of mothers’ groups is more similar to that of ASD. Some bacteria are shared between fathers, mothers, and ASD, but they are not the most abundant. It is well known that there is a relationship between gut microbiota and autism, so more studies like these are needed to fully understand and unravel the details of this relationship, which in turn will facilitate the development of probiotic and prebiotic interventions.

## Availability of Data and Materials

The data and materials used in this article are available with corresponding author.
